# Liquid–liquid phase separation-driven regulated cell death: from molecular mechanisms to therapeutic strategies

**DOI:** 10.1038/s41420-026-03150-7

**Published:** 2026-05-12

**Authors:** Bowen Li, Zhou Lan, Hao Cui, Wei Liu, Zhen Tian, Junxiang Lian, Yuyue Zhao, Guangtao Yu

**Affiliations:** 1 Stomatological Hospital, School of Stomatology, Southern Medical University, Guangzhou, China; 2 The Eighth Affiliated Hospital, Southern Medical University (The First People’s Hospital of Shunde, Foshan), Foshan, China

**Keywords:** Cell death, Diseases

## Abstract

Regulated cell death (RCD) is a tightly controlled biological process essential for development, tissue homeostasis, and host defense. Despite extensive investigation, how these signaling pathways are spatially and temporally organized to ensure precise cell death decisions remains incompletely understood. Liquid–liquid phase separation (LLPS), a biophysical mechanism driving the formation of dynamic biomolecular condensates, has emerged as a fundamental principle for cellular organization, signal integration, and stress adaptation. Increasing evidence indicates that LLPS plays a critical role in orchestrating cell fate decisions; however, its involvement in RCD has not yet been systematically defined. In this review, we provide a comprehensive overview of LLPS in the context of RCD. We summarize the physicochemical principles, molecular determinants, and regulatory factors governing LLPS, as well as the functional properties of phase-separated condensates, and then discuss how LLPS modulates key RCD pathways, including apoptosis, necroptosis, autophagy-dependent cell death, pyroptosis, and ferroptosis, highlighting shared and pathway-specific regulatory mechanisms. Furthermore, we examine the pathological consequences of aberrant phase separation–mediated RCD in human diseases and discuss emerging therapeutic strategies aimed at targeting LLPS-driven cell death processes. Finally, we catalog RCD-associated proteins with phase separation potential and outline major conceptual and technical challenges, proposing future directions for this rapidly evolving field.

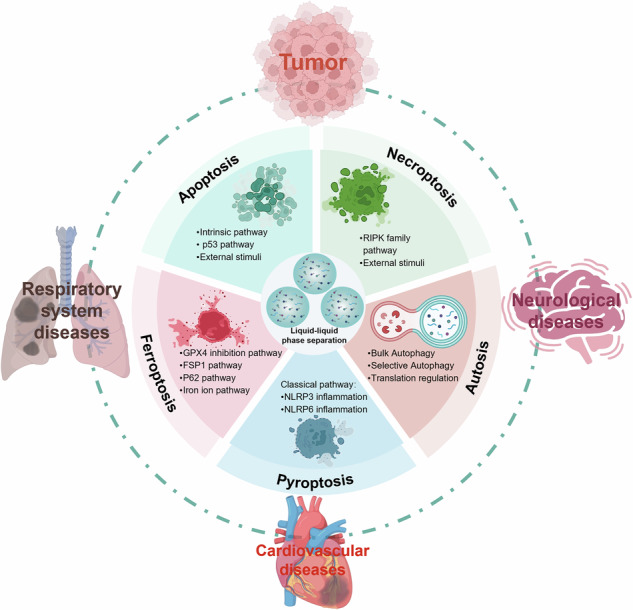

## Facts

- The phenomenon of liquid–liquid phase separation has been confirmed to play a role in various forms of regulated cell death.
- Certain molecules with the potential for phase separation have been identified as regulators of regulated cell death; direct evidence confirming the occurrence of phase separation in these contexts is still lacking.
- Research on liquid–liquid phase separation in regulated cell death remains a largely unexplored area.

## Open questions

- What are the underlying microscopic mechanisms that regulate the initiation and progression of liquid–liquid phase separation?
- How can liquid–liquid phase separation be harnessed to achieve precise and targeted regulation of regulated cell death?
- How the abnormal liquid–liquid phase separation regulated by regulated cell death mediates the occurrence of diseases?
- Do relative proteins with phase separation potential also participate in regulating programmed cell death through the phase separation mechanism?

## Introduction

Regulated cell death (RCD) is a core biological process that maintains tissue homeostasis, eliminates damaged cells, and defends against pathogen invasion [1, 2]. According to the mechanistic definition established by the NCCD in 2018, RCD is no longer confined to traditional apoptosis, but has expanded to include necroptosis, pyroptosis, ferroptosis, and autophagy-dependent cell death—each governed by relatively distinct signaling cascades and execution molecules [3]. Current studies reveal that RCD pathways do not operate independently but exhibit extensive crosstalk through shared key nodes, compensatory mechanisms, and bidirectional regulation. The same stimulus can trigger distinct death outcomes depending on cellular conditions, while individual molecules often function as hubs across multiple death pathways [4]. For example, caspase-8 inhibition can divert TNFR1 signaling from apoptosis to necroptosis [5]; meanwhile, the pyroptosis executor gasdermin D not only forms plasma membrane pores but also translocates its N-terminal fragment to mitochondria, inducing damage and amplifying crosstalk with the intrinsic apoptotic pathway [6]. This functional interconnectivity endows the RCD system with high plasticity and signal redundancy, thereby explaining why monotherapeutic blockade of a single pathway often fails to rescue cells under pathological conditions [7]. In summary, the essence of RCD lies not in “multiple parallel pathways” but rather in an interconnected network collectively supported by shared receptors, adapter proteins, proteases, kinases, and organelle stress platforms. Although key nodal proteins (e.g., RIPK1, caspases, MLKL, GSDMs) and their regulatory interactions within these pathways have been identified [8, 9], the molecular mechanisms that integrate these signals, coordinate them spatiotemporally, and ultimately determine the cell death outcome remain a major knowledge gap. Linear pathway analyses fall short in capturing this multidimensional signaling integration, underscoring the need for new conceptual frameworks and physical models to elucidate the decision-making logic of RCD.

In recent years, liquid–liquid phase separation (LLPS)—an emergent mechanism underlying the formation and functional regulation of membrane‑free organelles—has offered a paradigm-shifting perspective for understanding complex signal transduction processes [10, 11]. This process drives the formation of highly dynamic biomolecular condensates—exemplified by nucleoli, RNA polymerase II condensates, stress granules, and P-bodies—which enable spatiotemporally precise regulation of biochemical reactions through localized enrichment of reaction components, sequestration of inhibitory factors, or assembly of signaling platforms [12]. Notably, key proteins involved in diverse RCD pathways—including autophagy regulator p62, stress granule component G3BP1 engaged in apoptotic stress response, and pyroptosis-mediating ASC specks—have been demonstrated to undergo LLPS-driven biomolecular condensation [13, 14]. The formation and disassembly of such condensates may function as a molecular toggle, determining whether cell death signals are propagated or terminated—thereby governing the critical decision between initiating “silent” apoptosis or “explosive” inflammatory death.

Therefore, this review aims to systematically elucidate the formation and biological functions of phase separation, and discuss recent advances in LLPS within RCD pathways and related diseases. We propose that LLPS may act as a pivotal molecular switch in the RCD signaling network, whereby its condensates sense and integrate multiple input signals, thereby influencing and ultimately determining cellular fate decisions in complex networks. A deeper understanding of this mechanism will not only enrich our comprehension of the complexity of RCD regulation but also provide potential molecular targets and a theoretical foundation for interventions in diseases driven by RCD dysregulation, such as cancer, neurodegenerative disorders, and inflammatory conditions.

## Liquid–liquid phase separation

**Fig. 1:**
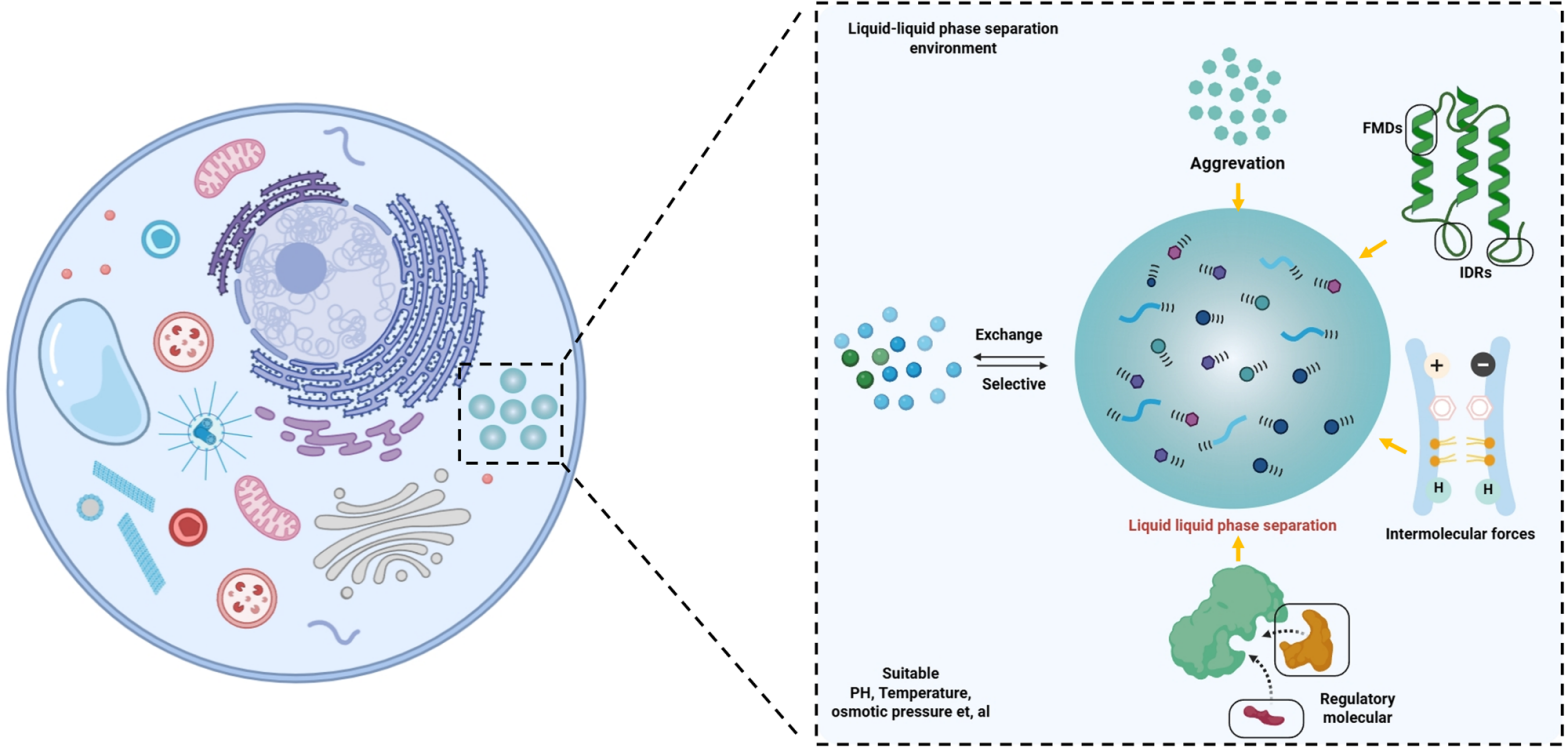
Intracellular LLPS condensation. The formation of intracellular phase-separated condensates is governed by multiple factors, including protein concentration, folded multivalent domains (FMDs), intrinsically disordered regions (IDRs), intermolecular interactions, molecular chaperones, and favorable environmental conditions. Within these condensates, molecules remain highly dynamic and can selectively exchange materials with the surrounding environment through specific mechanisms.

LLPS, a physiological process, leads to the formation of membraneless organelles, a phenomenon also recognized as LLPS. Upon the stimulation, weak non-covalent interactions drive the partitioning of the homogeneous intracellular environment into two distinct phases: a concentrated phase and a diluted phase, which differ significantly in their characteristics and functions [15]. In the concentrated phase, molecules form aggregates, leading to a higher molecular density and enhanced molecular activity, which facilitates more efficient material exchange [16]. In biology, the condensed phase is termed a biological condensation, which may also be referred to as an aggregate, mass, or particle (Fig. 1).

### The basic conditions

#### Concentration of biomolecules

The Flory-Huggins theory provides a comprehensive theoretical framework for understanding LLPS. In thermodynamic models, the formation of biomolecular condensates is an entropy-decreasing process. Consequently, in concentrated solutions, biomolecules must engage in more frequent and intimate interactions to compensate for the entropy loss associated with aggregation. The critical molecular concentration represents the threshold at which the additional free energy gained from interactions between concentrated and diluted phases balances the entropy loss due to condensate formation. Above this critical concentration, condensates emerge; below it, the solution remains homogeneous [15, 17]. Furthermore, in recent studies, the size and number of condensates exhibit a direct proportionality to the concentration of biomolecules: both the size and number of biomolecular condensates are observed to increase accordingly upon molecular concentration increases [18, 19]. Moreover, experimental observations of increased molecular concentration leading to changes in the number and size of condensates provide a robust method to verify whether LLPS occurs in living systems [20]. In conclusion, the concentration of biomolecules is a fundamental determinant for LLPS formation. Higher concentrations can enhance free energy, thereby compensating for the entropy loss associated with condensate formation to form phase-separated condensation.

#### Molecular characteristics

Currently, there are two key molecular characteristics facilitating the formation of phase-separated condensates: one is folded molecular domains (FMDs); the other is intrinsically disordered regions (IDRs) [15]. In multivalent systems, intracellular FMDs tend to undergo LLPS, which contains typical SRC homology 3 (SH3) domains and their proline-rich motif ligands. The higher the valency, the more pronounced the LLPS phenomenon becomes [15]. IDRs are referred to as low-complexity regions. Although they exhibit similar functional properties, FMDs are determined by molecular structure, whereas IDRs are defined by specific amino acid sequences within the molecule [16]. IDRs, promoting weak interaction to induce LLPS, are partly because molecules containing IDRs lack stable structures and exhibit more dynamic and variable conformations. Additionally, the disordered nature of these regions may facilitate increased binding affinity[17].In conclusion, IDRs can serve as one of the primary criteria for assessing whether a molecule is capable of undergoing LLPS. The evidence has shown that molecules containing IDRs are more likely to exhibit LLPS [18]. However, there is ongoing debate regarding whether IDRs alone are sufficient to induce LLPS. Therefore, more precise and reliable indicators are still required for accurate identification of LLPS events.

#### Intermolecular forces

The occurrence of LLPS requires intermolecular forces to drive the formation of aggregates, which can adopt a colloidal form. In this process, hydrogen bonds, van der Waals forces, and depletion forces contribute to colloidal aggregation [21]. Notably, van der Waals forces play a significant role in multivalent interactions and, together with Coulomb forces, facilitate the formation of phase-separated condensates [22]. Additionally, hydrophobic effects and cation-π interactions can facilitate low-affinity interactions and multivalent binding in specific regions of the molecule, thereby enhancing overall molecular affinity and promoting LLPS [23]. In addition to the aforementioned molecular forces, electrostatic interactions, and other intermolecular forces can promote the aggregation of specific molecules and serve as key drivers of LLPS.

#### Regulatory molecules

Regulatory molecules, including molecular chaperones and ligand pairs, play a crucial role in modulating LLPS. For instance, a member of the heat shock protein family, HSPB1, is a molecular chaperone and phased-separation regulator of cytoplasmic for TDP-43 [24]. Additionally, TNPO1, another molecular chaperone, regulates the LLPS of the RNA-binding protein FUS [23]. This phenomenon is primarily attributed to the variations in intermolecular forces. Ligands also exert regulatory effects on LLPS; for example, XS561 has been shown to modulate the LLPS behavior of Nur77 [25]. This mechanism is mainly reflected in the fact that ligands support the initiation of LLPS and facilitate LLPS in low-concentration proteins; conversely, unstable ligands can dissolve pre-formed condensates [26, 27].

In summary, LLPS is driven via molecular aggregation, intermolecular forces, intrinsic molecular structures, and the stimulation of molecular chaperones. In addition to the aforementioned conditions, environmental factors, via modulating the strength of intermolecular interactions and the solvation compatibility of molecules, play a crucial role in determining the occurrence of LLPS [28], including pH, osmotic pressure, co-solutes, temperature, and ionic strength, and significantly influence the formation of phase-separated condensates [12, 17]. In conclusion, these insights inspire a promising intervention strategy: the rational manipulation of phase separation—by leveraging its well-defined biophysical drivers—to precisely regulate fundamental biological processes.

### Non-RCD-related biological effects

LLPS initiates in a homogeneous environment, during which the initially uniform liquid undergoes phase transformation into concentrated and diluted phases. The internal molecules within the condensed phase, due to the compressive forces, exhibit high mobility and concentration, leading to the highly dynamic nature of the condensed phase. This manifests as rapid material exchange and dynamic reversibility [16, 17]. The phased separation condensates exhibit selective permeability, enabling them to enrich or exclude specific molecules [29]. The LLPS-based barrier of the nuclear pore complex in cells selectively permits the passage of small molecules, whereas large molecules require mediation by nuclear transport receptors for entry [30]. The formation of P-bodies and stress granules involves the selective condensation of RNA, thereby regulating RNA storage and degradation [31]. In summary, the selective permeability of these condensates enables precise intracellular compartmentalization and regulation of various biological processes within the cell.

Based on the above characteristics, LLPS forms membraneless organelles in cells with biochemical reaction rates faster than the surrounding medium, widely studied in the nucleus, cytoplasm, and cell membrane, and plays a role in maintaining genomic stability, transcription, cell signal transduction, and protein homeostasis [32]. The nucleolus is essentially a large-sized condensate formed by the phase separation of RNA and proteins, which regulates ribosome assembly and repairs misfolded proteins to maintain cellular homeostasis[33]; Nuclear RNA polymerase (Pol) II undergoes phase separation with various factors carrying IDRs, such as transcription factors and co-activators, to form condensates, which are not only present at the promoter sites but also at the super-enhancers composed of enhancer clusters, determining the transcription of key mRNAs and multiple non-coding RNAs and thus influencing cell fate [34]；P-bodies, which are formed by the phase separation and condensation of RNA and proteins in the cytoplasm, serve as key sites for mRNA storage and degradation, meanwhile abnormal condensation of P-bodies is associated with the occurrence and development of diseases, including cancer and neurodegenerative disorders [29]；After the T cell receptor on the cell membrane is activated, it undergoes multivalent binding through its own SH2 and SH3 domains or downstream proteins containing SH2 and SH3 domains and PRM proteins, causing phase separation and subsequently activating downstream reactions to lead to T cell activation [16]. In summary, phase-separated condensates exhibit a functional duality: they maintain cellular homeostasis under physiological conditions yet can drive pathological dysregulation when perturbed—thereby acting as pivotal determinants of cell fate. Consequently, elucidating the mechanistic pathways through which phase separation governs diverse biological processes, especially in the aspect of RCD, offers a rational foundation for harnessing it as a tunable regulatory switch in therapeutic intervention.

## Liquid–liquid phase separation in regulated cell death

In biological processes, RCD refers to a cell death mechanism mediated by molecular programs regulated by specific genes within the cell. Given the diversity of death modes and the complexity of the associated signaling pathways in RCD, it is essential to explore its underlying mechanisms from a more microscopic perspective. Studies have demonstrated that LLPS, an intracellular phenomenon involving molecular aggregation, plays a critical role in regulating RCD, serving as a dynamic molecular platform that recruits key signaling molecules to either promote or suppress their effector functions, akin to a switch that controls RCD initiation and thereby modulates cell fate [4], including apoptosis, necroptosis, autophagy-dependent cell death, pyroptosis, and ferroptosis

### Apoptosis

#### Intrinsic pathway

**Fig. 2:**
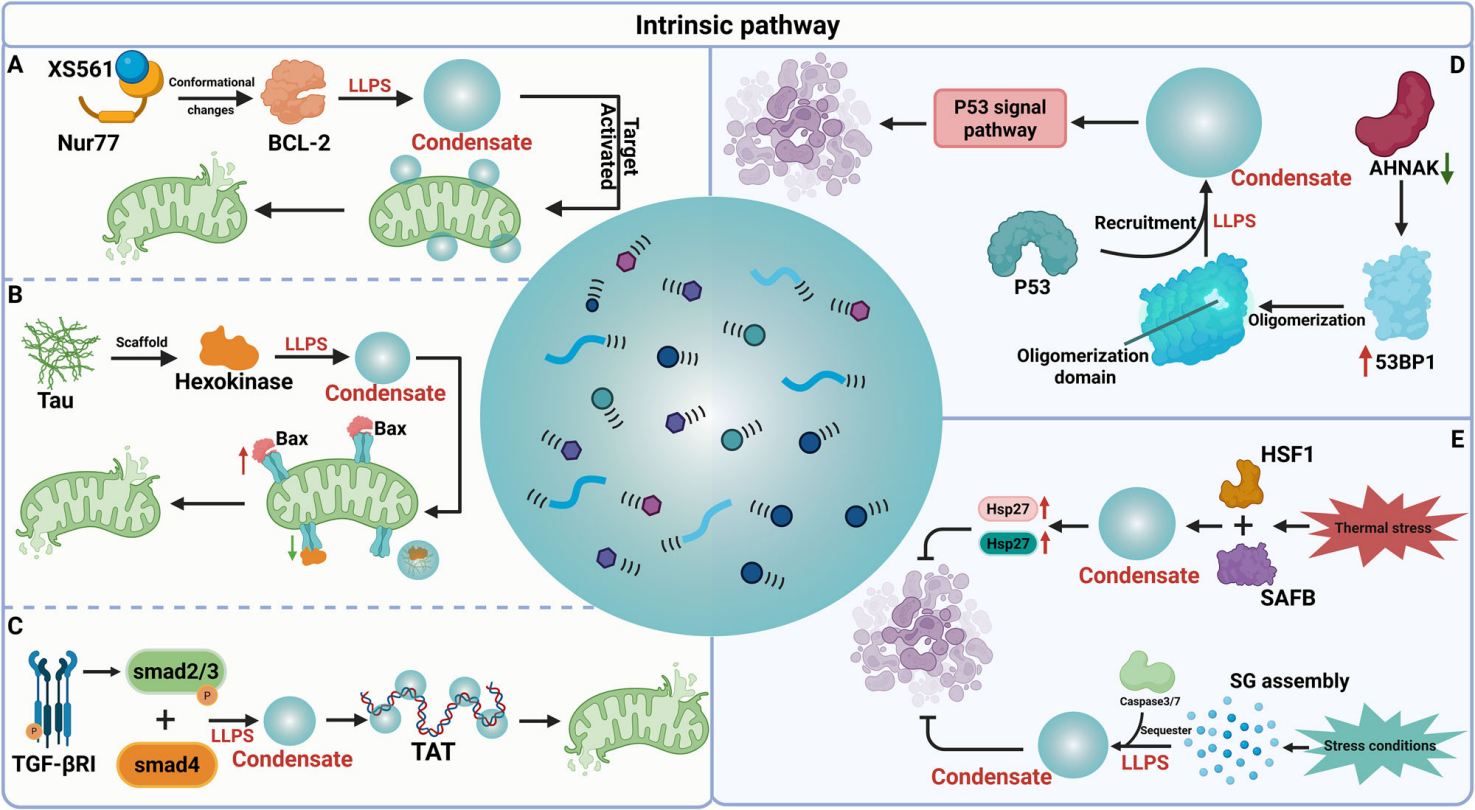
LLPS in several pathways of apoptosis. The text highlights that within intrinsic apoptosis: **A** XS561 triggers Nur77 LLPS, inducing BCL2 conformational changes and subsequently promoting apoptosis. **B** Tau44 undergoes LLPS to sequester hexokinase, thereby enhancing apoptosis. **C** Smd2/3/4 condensates enhance TAT translation and contribute to apoptosis induction. **D** AHNAK depletion promotes 53bp1 LLPS, facilitating the recruitment of p53 to mediate apoptosis. **E** Under external stress, the interaction between HSF1 and SAFB drives LLPS and SGs condensates sequesters caspase3/7 to exert anti-apoptotic effects.

Classical apoptosis in RCD is primarily regulated by the well-established intrinsic pathway and extrinsic pathway [4]. The intrinsic pathway is linked to outer mitochondrial membrane permeabilization (OMMP(2)) and is regulated by the Bcl-2 family of proteins under normal conditions to prevent apoptosis [35]. Upon receiving external stimuli, the BH-3-only proteins of the Bcl-2 family undergo conformational changes and insert into the outer mitochondrial membrane (OMM), promoting OMMP, which leads to the release of mitochondrial proteins, such as cytochrome c and Smac/DIABLO [36]. Cytochrome c in the cytoplasm will bind to Apaf-1 and, with the participation of dATP/ATP, assemble into an apoptosome; the complex recruits and activates caspase-9, which in turn activates the effector caspases, mainly caspase-3 and caspase-7; meanwhile, Smac/DIABLO can neutralize the inhibition of caspases by IAP proteins such as XIAP. Eventually, the activated effector caspases will cleave a large number of cellular substrates, leading to typical apoptotic phenotypes: chromatin condensation, DNA fragmentation, cell shrinkage, and membrane blebbing [37]. As an orphan nuclear receptor, Nur77 undergoes LLPS via its IDRs to form condensates upon the stimulus of ligand XS561. The condensation exits the nucleus and targets the BCL-2 loop structure on mitochondria, inducing conformational changes that convert Bcl-2 from a survival factor to a pro-apoptotic agent in tumor cells. Additionally, XS561 phosphorylates Bcl-2, promoting LLPS of the Bcl-2 loop region, which facilitates the formation of Nur77/Bcl-2 condensates, then promotes the release of cytochrome C, the binding of Apaf-1, the formation of apoptosomes, and the activation of Caspase, ultimately leading to Nur77/Bcl-2-mediated intrinsic pathway apoptosis in tumor cells (Fig. 2A) [25]. In Alzheimer’s disease, the tubulin-associated unit (Tau)-441 protein can undergo LLPS to sequester hexokinase (HK), thereby reducing the levels of free HK in the cytoplasm. HK competes with Bax, a member of the Bcl-2 family, for binding to the voltage-dependent anion channel 1 (VDAC1) on the mitochondrial membrane [38]. The isolation of HK via condensation increases Bax binding to VDAC1, promoting the occurrence of the above-mentioned BCL-2 family-mediated apoptosis. Conversely, terminating Tau-441 LLPS significantly inhibits this apoptotic process (Fig. 2B) [39]. In hepatocellular carcinoma, phosphorylated TGF-βRI within TGF-β signaling pathway phosphorylates and activates Smad2/3, which form a heterodimer to recruit Smad4, a protein with phase-separation capability, to form a trimeric complex, which migrates into the nucleus and forms phased separation condensation and binds to the tyrosine aminotransferase (TAT) gene to enhance its transcription, leading to elevated levels of reactive oxygen species (ROS) within mitochondria [40]. Ultimately, mitochondrial damage results in the release of cytochrome C into the cytoplasm, activating Caspase-9 and triggering intrinsic apoptosis (Fig. 2C) [40, 41]. p53, a well-characterized tumor suppressor gene, regulates intrinsic apoptosis by promoting the upregulation of Bcl-2 family proteins [42]. In the absence of AHNAK (a scaffold protein that competes with p53 for the OD domain of 53BP1), 53BP1 undergoes oligomerization and LLPS to form condensates, which subsequently bind to p53 and promote apoptosis in tumor cells (Fig. 2D). Interestingly, in normal cells, this process leads to a milder outcome, resulting in cellular senescence [43]. In addition, to respond to external stimuli, cells will undergo a series of physiological reactions, among which the occurrence of intrinsic apoptosis is included [44]. Upon thermal stress, heat shock transcription factor 1 (HSF1) and scaffold attachment factor B (SAFB) within nuclear stress bodies (nSBs) undergo LLPS, leading to the upregulation of Hsp27 and Hsp70, thereby enhancing cellular thermotolerance. Conversely, disrupting this LLPS, for instance by using 1,6-hexanediol (a well-established phase separation inhibitor [45]), enhance the sensitivity of cells to heat stimulation, thereby triggering a series of endogenous apoptotic cascade signals, including Bax/Bak-related mitochondrial outer membrane permeabilization, cytochrome c release, apoptosome formation, and Caspase activation [46] (Fig. 2E). In summary, these findings collectively demonstrate that LLPS plays a pivotal role in regulating intrinsic apoptosis, with most condensates forming under ligand stimulation. Therefore, identifying key ligands associated with apoptotic proteins and predicting the structural features of IDR could significantly enhance our ability to determine whether LLPS in these proteins modulates intrinsic apoptotic pathways.

#### Extrinsic pathway

The extrinsic pathway is primarily initiated by the interaction between cell death ligands and their receptors, leading to the formation of a death-inducing signaling complex (DISC), which subsequently activates caspase-8 and caspase-10 to trigger apoptosis [47]. TNF-related apoptosis-inducing ligand (TRAIL), a cell death ligand, has been demonstrated to promote apoptosis by inducing LLPS through liposome-mediated formation of TRAIL clusters [48]. Although it has not yet been reported whether other ligands, such as TNF-α and Fas, undergo LLPS to regulate extrinsic apoptosis, the previous discussion on ligand-mediated LLPS and its role in cell signaling transduction [16] suggests that LLPS may play additional functions in extrinsic apoptosis. This warrants further investigation into the potential involvement of LLPS in the regulation of extrinsic apoptotic pathways.

In conclusion, previous studies have revealed that phase-separated condensates form under stress and thermal stimuli, exerting regulatory effects on apoptosis. Given that LLSP itself constitutes an entropy-reducing process, a critical question arises: External stimuli may potentially compensate for the entropy reduction process, thereby facilitating the aggregation and condensation of biomolecules. Stress granules (SGs) are dynamic, phase-separated condensates that form in the cytoplasm of cells in response to environmental stress [49]. During apoptosis induced by external stimuli, SGs sequester executioner caspase-3/7 (pro-caspase3/7), thereby preventing their activation into active caspase3/7 and promoting cell survival under short-term stress conditions [50]. If external pressure can serve as a condition for LLPS, we could utilize this condition to (1) stimulate the formation of biomolecular LLPS and employ it as a means to investigate the properties of condensation, and (2) more importantly, control LLPS formation through this approach. Such controlled regulation would enable precise manipulation of biological processes, particularly in contexts such as inflammation, cancer progression, and metabolic growth.

### Necroptosis

Necroptosis is a gene-regulated form of lytic RCD primarily controlled by death receptors, which, along with their associated kinases, determine the ultimate fate of the cell, either apoptosis or necroptosis [51]. Current research indicates that necroptosis is primarily mediated by the RIPK family, which plays a central role in the signaling cascade: upon TNF-α activation, membrane-bound TNFR1 is activated and recruits RIPK1, which blocks downstream NF-κB activation and apoptotic signaling; subsequently, activated RIPK1 interacts with its homologous protein RIPK3 to form a necrosome complex, where RIPK3 phosphorylates mixed lineage kinase domain-like protein (MLKL), promoting MLKL oligomerization and its binding to phosphatidylinositol phosphates (PIPs) on the inner leaflet of the plasma membrane, ultimately inducing necrotic plasma membrane permeabilization and triggering necroptosis execution [51–54]. In this pathway, the PARylation-dependent ubiquitination (PARdU) complex and the E3 ubiquitin ligase RING finger protein 146 (RNF146), forms a functional unit; subsequently, TAX1BP1 acts as an adapter protein to mediate the recruitment of this complex to RIPK3, facilitating the formation of LLPS condensate, which promotes the proteasomal degradation of activated RIPK3, thereby inhibits the binding of MLKL to PIPs and then reduces the permeability of the necrotic cell membrane and hindering necroptosis [55]. Intriguingly, one of the reasons for this condensation formation is that kinase-activated RIPK1 oligomerizes, which increases the local concentration of these proteins. In addition, under oxidative stress conditions, SGs form phase-separated condensates that serve as platforms to anchor Z-RNA [56]. Subsequently, the condensation recruits Z-RNA and induces ZBP1 recognition to Z-RNA, following active ZBP1 recruits RIPK3 within the SGs to form necrosomes, ultimately leading to the initiation of necroptosis [57].

In conclusion, phase-separated condensates function as isolation compartments that sequester necrosis-related proteins, thereby suppressing their activity and inhibiting the activation of downstream signaling pathways. This mechanism effectively exerts negative regulation on necroptosis. Consequently, these findings imply the potential application of condensates as inhibitors for pathogenic proteins by either leveraging their competitive effects or promoting the degradation of target proteins, ultimately preventing disease progression. Furthermore, it was observed that LLPS frequently occurs subsequent to protein oligomerization. This phenomenon is hypothesized to arise because oligomerization enhances both the effective molecular concentration and the capacity for multivalent interactions, consequently lowering the critical concentration threshold required for LLPS. As previously discussed, the oligomerization of RIPK1 increases the molecular density within condensates. For instance, proteins containing low-complexity domains (LCDs) are more prone to induce LLPS via multivalent interactions following oligomerization [10]. Another possible explanation is that oligomerization modulates the aggregation energy of molecules, thereby affecting the thermodynamic equilibrium of LLPS [58]. For example, specific RNA-binding proteins, such as FUS and TDP-43, exhibit enhanced adhesive properties following oligomerization, which contributes to the stabilization of condensates formed via LLPS. In necroptosis, MLKL is predicted to possess IDR structures conducive to phase separation (Phaspre), and its functional activation requires both oligomerization and binding to PIPs. Whether this binding mechanism involves LLPS as a critical regulatory step merits further in-depth investigation.

### Autophagy-dependent cell death

Autophagy-dependent cell death is initiated when excessive activation of autophagic signaling pathways leads to a shift in the autophagic process from maintaining cellular homeostasis and survival to promoting RCD [59]. Functionally, it was validated to occur during the development of specific organs in mammalian embryos and the degeneration of salivary glands in Drosophila melanogaster as a form of developmental cell death [60]; mechanistically, it is observed when apoptotic signaling pathways, such as those involving BAX and BAK, are inhibited [61, 62].

Given that the progression of autophagy-dependent cell death is dependent on autophagy, the link between autophagy and LLPS has been extensively documented [63]. Thus, understanding the connection between autophagy and LLPS can provide deeper insights into the mechanisms of autophagic cell death. Current research indicates that LLPS events are predominantly observed in both bulk autophagy and selective autophagy [64].

#### Bulk autophagy

**Fig. 3:**
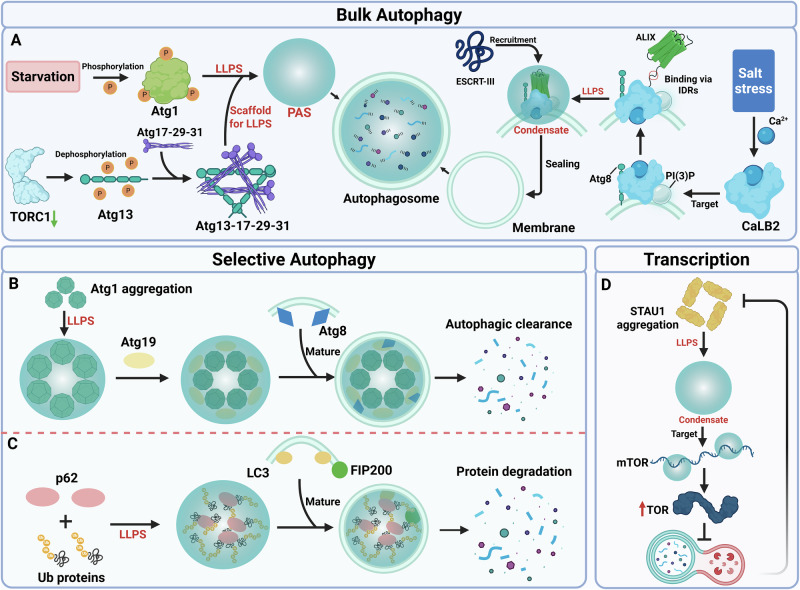
LLPS in several pathways of autophagy. **A** In macroautophagy, the ATG family proteins aggregate and undergo LLPS under starvation conditions to form PAS. During autophagosome maturation, the CaLB2/ATG8/PI(3)P complex on the autophagosomal membrane interacts with ALIX, driving LLPS and condensation that stimulate ALIX fiber assembly and promote ESCRT-III recruitment and localization, thereby facilitating autophagosome maturation. **B** In selective autophagy, Ape1 undergoes self-phase separation, enabling the recruitment and assembly of other Atg family proteins and ultimately driving autophagosome formation. **C** Additionally, p62 binds to ubiquitinated proteins and undergoes LLPS to form condensates, which directly interact with FIP200 to recruit additional autophagy signaling molecules and promote autophagosome formation. **D** In terms of nucleic acid modification, STAU1 undergoes LLPS to form biomolecular condensates that target mTOR, which promotes its activation and translation, enhancing mTOR signaling and leading to the inhibition of autophagy.

The initial step in bulk autophagy is the formation of the pre-autophagosomal structure (PAS), which begins following the assembly of the Atg1 complex in yeast, which is homologous to the ULK1 complex in mammals [65]. In yeast, starvation-induced low TORC1 activity leads to reduced phosphorylation of Atg13 by PP2C phosphatase to recruit Atg17-29-31; meanwhile, starvation activates Atg1 autophosphorylation. Subsequently, the Atg13-Atg17-29-31 complex acts as a protein scaffold to recruit Atg1 to form phased separation condensation—the early pre-autophagosomal structure (PAS). The autophosphorylated Atg1 then interacts with downstream factors and VAC8 to mature the PAS. Finally, the isolation membrane, also known as the phagophore, wraps around to form an autophagosome, which mediates autophagy (Fig. 3A) [66]. During the subsequent maturation stage of autophagosomes, the endosomal sorting complex required for transport (ESCRT) is essential for sealing the isolation membrane. Specifically, CaLB2 acts as a pioneer protein by binding ATG8 and phosphatidylinositol 3-phosphate (PI(3)P) through its C2 domain, thereby localizing to the autophagosome membrane. Then, CaLB2 interacts with the IDRs of ALIX (a conserved ESCRT-associated protein) via its IDRs, which triggers the formation of phased separation condensation. This condensate stimulates the assembly of ALIX filaments to promote the recruitment and localization of ESCRT-III. Ultimately, ESCRT-III seals the phagophore, facilitating the maturation of the autophagosome (Fig. 3A) [67]. The above results confirm that LLPS is present throughout the process of macroautophagy. Macroautophagy and selective autophagy represent the two major autophagic pathways; elucidating the role and molecular mechanisms of LLPS in macroautophagy—particularly in the context of selective cargo recognition, autophagosome biogenesis, and maturation—provides novel mechanistic insights into autophagy-dependent cell death.

#### Selective autophagy

Selective autophagy (SA) typically forms autophagosomes specifically around targeted cargoes [68]. This process has been demonstrated by research to involve LLPS. Here, we illustrate this phenomenon using Ape1 and p62 as examples of selective autophagy targets.

Following activation, Ape1 undergoes self-phase separation to assemble into a tetrahedral-shaped dodecamer, which is further interconnected through propeptide trimerization to form a condensate termed the Ape1 complex; subsequently, this condensate is recognized by Atg19 to recruit and assemble other Atg-family proteins, during which the continuous increase in Atg1 concentration triggers its phosphorylation, ultimately driving autophagosome formation (Fig. 3B) [69–71].

In addition, the selective autophagy receptor p62, also known as Sequestosome 1 (SQSTM1), facilitates the degradation of aggregated ubiquitinated proteins via LLPS [13]. p62 undergoes oligomerization on its own, then binds to ubiquitinated proteins and undergoes LLPS to form condensation [13]. These condensations directly interact with FIP200, recruiting other autophagic signaling molecules to facilitate the formation of autophagosomes, which subsequently migrate and undergo digestion [72]. Due to its inherent oligomerization capability, p62 exhibits enhanced activity and is more prone to undergo LLPS upon activation (Fig. 3C) [73]. Collectively, these findings suggest that molecular oligomerization not only facilitates LLPS but also serves as a tunable regulatory mechanism: by engineering oligomerization propensity, cells—or researchers—can drive condensate formation to spatiotemporally control key biological processes. This principle thus represents a promising and mechanistically grounded avenue for future investigation.

In addition to the aforementioned mechanisms, the autophagy-inhibiting mTOR pathway is also influenced by LLPS. The double-stranded RNA-binding protein Staufen1 (STAU1), which regulates RNA metabolism and thereby modulates physiological and pathological conditions, primarily localizes to SGs in the cytoplasm; upon excessive accumulation, STAU1 undergoes LLPS to form biomolecular condensates that target mTOR, which promotes its activation and translation, enhancing mTOR signaling and leading to the inhibition of autophagy (Fig. 3D) [74, 75].

Tripartite motif (TRIM) family proteins function as E3 ligases and are involved in various biological processes [76]. Among these, the coiled-coil (CC) domain of TRIM21 undergoes self-LLPS, promoting the formation of LC3 (autophagic membrane-associated proteins) condensation, which facilitates the assembly of autophagosomes, thereby mediating autophagy [77].

The above research highlights the critical role of LLPS in autophagy. As an emerging and increasingly recognized mechanism for regulating biological processes, investigating autophagy through the lens of LLPS offers a novel perspective to deepen our understanding of autophagic activities. Since autophagy-dependent cell death, it is evident that the interplay between LLPS and autophagy-dependent cell death warrants further exploration. This remains a relatively understudied field. Elucidating the function of LLPS in autophagy-dependent cell death could pave the way for innovative approaches to studying embryonic development, unraveling the pathogenesis, and improving therapeutic strategies for Alzheimer’s disease, as well as inhibiting protective autophagy to enhance autophagy-dependent cell death-based anti-tumor therapies.

### Pyroptosis

**Fig. 4:**
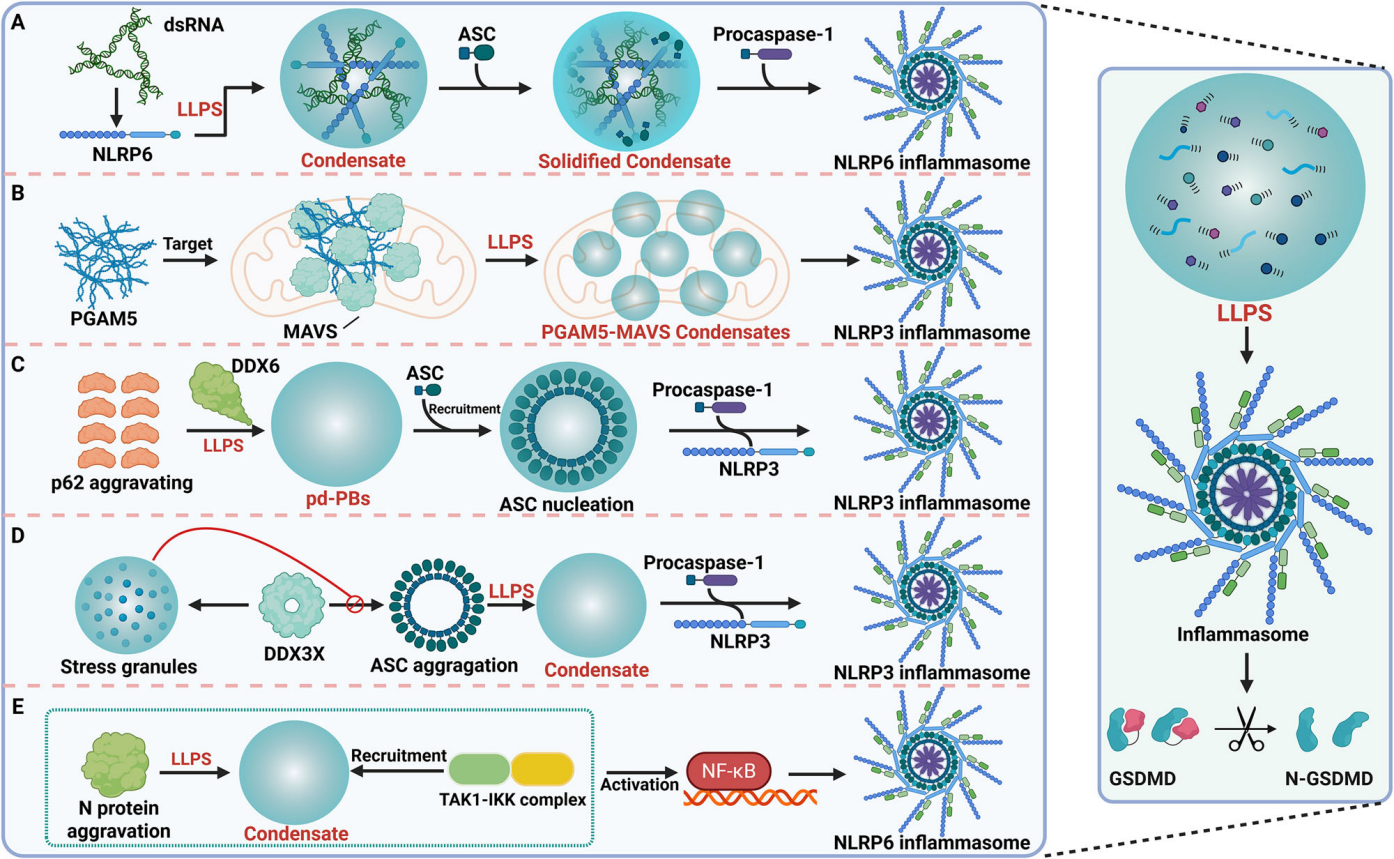
LLPS in pyroptosis. **A** dsRNA induces the LLPS of NLRP6, facilitating the recruitment of ASC into the condensation for pro-caspase-1 assembly, which ultimately leads to the formation of mature inflammasomes. **B** PGAM5 serves as a scaffold protein for MAVS, driving the formation of phase-separated condensation and enhancing the recruitment and assembly of NLRP3 inflammasomes. **C** Sequestosome 1/p62 aggregates and recruits DDX6, promoting its LLPS to form pd-pBs, and subsequently recruits associated molecules for inflammasome assembly. **D** LLPS of DDX3X competes between stress granules and the NLRP3 inflammasome, forming a rheostat-like regulatory mechanism that determines the survival or pyroptosis of cells under stress conditions. **E** N protein can undergo phase separation with TAK1 and IKK, concurrently suppress stress granule formation, promote NF-κB activation, and ultimately trigger cellular pyroptosis.

Pyroptosis is a type of RCD associated with inflammation, characterized by the formation of pores in the cell membrane, leading to cell membrane rupture and subsequent cell swelling, ultimately resulting in the release of intracellular contents, which often include pro-inflammatory factors [78, 79]. Current research on the interplay between LLPS and pyroptosis is predominantly centered on the classical pathway [80]. The classical pathway is the first discovered and most common pyroptosis pathway, characterized by the formation of inflammasomes. This pathway involves inflammatory caspases, intracellular pattern recognition receptors (PRRs), apoptosis-associated speck-like protein containing a caspase recruitment domain (ASC), and pro-caspase-1 [81]. Among the PRRs, nucleotide-binding oligomerization domain-like receptors (NLRs) [81] can promote the self-cleavage and activation of pro-caspase-1 to form caspase-1 by directly interacting with the C-terminal caspase recruitment domain (CARD) of pro-caspase-1 or through the adapter protein ASC. Subsequently, caspase-1 cleaves GSDMD, releasing the GSDMD-NT fragment, which induces pore formation, cell swelling, and ultimately pyroptosis [82]. During this process, dsRNA as a direct and potent ligand for NLRP6 promotes the LLPS of oligomerized NLRP6, leading to the formation of condensation, which increases the local concentration of the pyrin domain (PYD), facilitating filamentous PYD-PYD interactions that recruit ASC; subsequently, ASC recruits caspase-1 via filamentous CARD-CARD interactions, which solidifies the condensation and reduces their fluidity, ultimately resulting in the formation of mature inflammasomes (Fig. 4A) [83]. In addition, Zhang et al. discovered that phosphoglycerate mutase family member 5 (PGAM5) functions as a scaffold protein for mitochondrial antiviral signaling protein (MAVS), inducing the formation of phased separation condensation, which is characterized by a sponge-like structure, facilitating the recruitment of NLRP3, thereby promoting inflammasome activation (Fig. 4B) [84]. Meanwhile, Sequestosome 1/p62 condensation mentioned earlier can switch from selective autophagy function to inflammatory function under stress conditions [85]. In this situation, p62 undergoes aggregation, and the aggregates recruit Dead-Box (DDX6) (an ATP-dependent RNA helicase) and promote its LLPS, and then DDX6 can intensify the formation of p62 aggregates and facilitate their transformation into p62-dependent p-bodies (pd-pBs) which act as a platform to recruit the inflammasome structure APC and nucleate, ultimately recruiting other structures NLRP6 and its caspase-1 and nucleating again to form mature inflammasomes (Fig. 4C) [85]. Within the same family, DDX3X has been shown to promote the phase transition of ASC through its scaffold function, thereby activating the NLRP3 inflammasome [86]; additionally, its catalytic activity regulates the LLPS of stress granules, which competes with the inflammasome for DDX3X to inhibit pyroptosis [87]. In this process, DDX3X competes between stress granules and the NLRP3 inflammasome, forming a rheostat-like regulatory mechanism that determines the survival or death of cells under stress conditions (Fig. 4D) [86, 88]. Meanwhile, Lan et al. found that the N protein can undergo phase separation with TAK1 and IKK, concurrently suppress stress granule formation, promote NF-κB activation, and ultimately trigger cellular pyroptosis (Fig. 4E) [89].

In summary, research has demonstrated that the regulatory role of LLPS in the assembly of inflammasomes during pyroptosis (Fig. 4). Consequently, moderate LLPS facilitates the orderly assembly of inflammasomes, triggering appropriate local inflammatory responses to eliminate pathogens. In contrast, abnormal LLPS results in excessive inflammasome activation, thereby initiating pathological cascades. Therefore, exploring how to control the pyroptosis switch via LLPS warrants further investigation. Additionally, leveraging the physical and chemical properties of LLPS condensates (e.g., viscoelasticity, local molecular concentration, osmotic pressure, and ligand involvement) could enable more precise regulation of the pyroptosis threshold.

### Ferroptosis

Ferroptosis, an emerging form of RCD, has introduced significant advancements and novel perspectives in the field of cellular metabolism research and maintains cellular homeostasis as well as tumor suppression [90]. Although ferroptosis also involves cell death characterized by cellular swelling and rupture, it fundamentally differs from pyroptosis in that its primary cause is the excessive accumulation of lipid peroxides and its main objective is to maintain tissue homeostasis [91]. The primary pathways of ferroptosis, including GPX4 inhibition, FSP1 suppression, the Fenton reaction, and p62-mediated regulation of ferroptosis, are all closely associated with LLPS processes.

#### GPX4 pathway

**Fig. 5:**
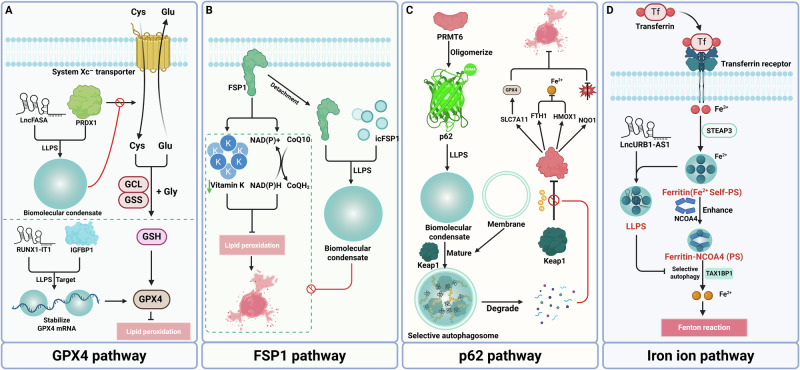
LLPS in several pathways of ferroptosis. **A** In the GPX4 inhibition pathway, LncFASA induces and promotes the LLPS of PRDX1, thereby inhibiting the regulation of SLC7A11 and GPX4 and inducing ferroptosis; RUNX1-IT1 binds to IGF2BP1 to undergo LLPS, which enhances GPX4 expression and prevents ferroptosis. **B** In the FSP1 pathway, icFSP1 binds to FSP1 to induce LLPS, inhibiting its function, ultimately inducing ferroptosis. **C** In the P62 pathway, PRMT6 promotes p62 oligomerization and LLPS, which recruit and sequester Keap1, thereby enhancing Nrf2 activity to inhibit ferroptosis. **D** In the iron ion pathway, reduced Fe^2+^ undergoes self-phase separation to form Fe^2+^ storage compartments. Then, NCOA4 dimerizes and interacts with ions to further form condensation, stabilizing the ion warehouse. During ferroptosis, TBX1TP1 induces the degradation of condensation, releasing Fe^2+^ ions that participate in the Fenton reaction, leading to ferroptosis. Additionally, URB1-AS1 binds Fe^2+^ via undergoing LLPS to compete with NCOA4, ultimately inhibiting ferroptosis.

GPX4 is a critical enzyme that prevents the accumulation of lipid peroxides in cells, thereby inhibiting the onset of ferroptosis [92]. The synthesis of GPX4 begins with cystine being transported into cells via the system Xc⁻ transporter, a heterodimer of SLC7A11 and SLC3A2 embedded in the cell membrane, followed by intracellular conversion of cystine to cysteine, which combines with glutamate and glycine to form glutathione (GSH) which serves as the substrate for GPX4, a selenoprotein that utilizes its intrinsic GSH to reduce cytotoxic lipid hydroperoxides into non-toxic lipid alcohols, thereby inhibiting ferroptosis [93, 94]. During this process, long non-coding RNA- LncFASA binds to PRDX1, which regulates the expression of SLC7A11 and GPX4 and promotes its LLPS. The LLPS of PRDX1 inhibits its activity, leading to reduced expression of SLC7A11 and GPX4, thereby resulting in intracellular lipid peroxidation and inducing ferroptosis (Fig. 5A) [95]. In addition, long non-coding RNA-RUNX1 intronic transcript 1 (RUNX1-IT1) inhibits ferroptosis by enhancing GPX4 expression through LLPS. Specifically, RUNX1-IT1 binds to IGF2BP1, an N6-methyladenosine (m^6^A) reader, and promotes its phase separation. The resulting condensates increase the occupancy of IGF2BP1 on mGPX4 mRNA, stabilizing its structure and thereby enhancing GPX4 expression, ultimately facilitating the degradation of lipid peroxides and preventing ferroptosis (Fig. 5A) [96]. Similarly, in the context of transcriptional regulation, the transcriptional coregulators YAP and TAZ can form phase-separated condensates upon Mn^2+^ induction [97]. These condensates subsequently translocate into the nucleus and target long-chain acyl CoA synthetase 4 (ACSL4) and promote its expression level, which enhances PUFA (a key step in ferroptosis execution [98]) peroxidation by ACSL4, ultimately activating ferroptosis [97]. It is evident that LLPS not only enhances the activity and functionality of proteins in necroptosis but also exerts a silencing effect during ferroptosis. This suggests that for certain key proteins involved in ferroptosis, controlling the conditions of LLPS could enable us to modulate LLPS as a switch, thereby regulating the progression of ferroptosis.

#### FSP1 pathway

Ferroptosis suppressor protein 1 (FSP1), an oxidoreductase, functions as a parallel alternative to GPX4 [99]. Through its NADH-dependent quinone oxidoreductase activity, FSP1 reduces ubiquinol to ubiquinone (coenzyme Q) and also reduces vitamin K. The reduced forms of CoQ and vitamin K can scavenge lipophilic free radicals, thereby preventing lipid peroxidation and ferroptosis. Given that the ferroptosis inhibitory effect mediated by FSP1 is independent of GPX4, it is considered the second major system for protecting cells against ferroptotic cell death [99, 100]. Recent studies have confirmed that the inhibitory effect of icFSP1 on FSP1 primarily depends on LLPS. Mechanistically, icFSP1 interacts with FPS1’s specific residues (S187, L217, and Q319) within IDR, thereby reducing FSP1’s affinity for the membrane and promoting formation of phased separation condensation to silence FSP1 and prevent reduction of ubiquinone and vitamin K, ultimately inducing ferroptosis (Fig. 5B) [101]. The research demonstrates that the material regulates ferroptosis via LLPS, thereby modulating biological activities. Likewise, relevant experiments confirm that LLPS can serve as a controllable method to regulate the localized effects of drugs [102]. Consequently, employing drug-induced LLPS to control the modes of ferroptosis may offer a novel strategy for investigation.

#### p62 pathway

The regulation of ferroptosis by p62 primarily depends on the autophagy mechanism described earlier. Specifically, P62 inhibits Keap1-dependent degradation of Nrf2, thereby stabilizing Nrf2, a key regulator that counteracts ferroptosis, and leading to reduced levels of free iron and increased synthesis of GSH, ultimately mitigating ferroptosis [103, 104]. Mechanistically, protein arginine methyltransferase 6 (PRMT6) promotes the oligomerization of p62 via asymmetric dimethylarginine (ADMA) modification, thereby inducing p62 LLPS and the formation of p62 condensates. These p62 condensates recruit and sequester Keap1, facilitating its degradation and inhibiting Keap1-mediated Nrf2 degradation. Consequently, this enhances the Nrf2 signaling axis and suppresses ferroptosis (Fig. 5C) [105]. This further corroborates that protein oligomerization and intrinsic disorder enhance the likelihood of LLPS. The resulting condensation functions as a biomolecular organelle, facilitating normal physiological activities and orchestrating programmed biological processes, thereby contributing significantly to the maintenance of cellular homeostasis.

#### Iron ion pathway

In addition to the primary mechanisms of ferroptosis described above, the ionic mechanism also plays a role in this RCD. The Fenton reaction, which utilizes hydrogen peroxide and labile iron as substrates, generates hydroxyl radicals (·OH) and peroxyl radicals (ROO·) upon reaction, which induce lipid peroxidation, thereby promoting ferroptotic cell death [106]. Mechanistically, after trivalent iron ions (Fe^3+^) are reduced to divalent iron ions (Fe^2+^), they undergo self-phase separation to form storage compartments for Fe^2+^ via metal reductase STEAP [107, 108]. During this process, nuclear receptor coactivator 4 (NCOA4) dimerizes and interacts with Fe^2+^ ions to form phase-separated condensates, thereby promoting the formation of these iron-rich condensates; subsequently, during ferroptosis, TBXTP1-induced condensates are engulfed and degraded, releasing Fe^2+^ ions that participate in Fenton reactions, leading to the accumulation of reactive oxygen species (ROS) and lipid peroxides and promoting lipid peroxidation to induce ferroptosis(Fig. 5D) [109]. In addition, long non-coding RNA-URB1-AS1 competes with NCOA4 for divalent iron ions (Fe^2+^). Its internal secondary structure binds to Fe^2+^, leading to LLPS and the formation of condensates. In contrast to NCOA4, these URB1-AS1-induced condensates inhibit iron ion autophagy, thereby inhibiting the occurrence of ion reactions mediated by it to reduce the content of peroxides to suppress ferroptosis (Fig. 5D) [110].

In summary, these findings indicate that autophagy can modulate ferroptosis via LLPS. This crosstalk between distinct cell death modalities warrants further investigation. Moreover, autophagy and apoptosis are capable of interacting in a context-dependent manner, either promoting or antagonizing each other [4]. This offers two insights: first, identifying key proteins involved in cross-phase separation and validating their phase separation capabilities may enable more precise regulation of RCD; second, leveraging these cross-phase separation proteins could enhance the synergistic or inhibitory interactions among various forms of RCD, thereby contributing to the maintenance of cellular homeostasis and aiding in disease prevention and treatment.

## Liquid–liquid phase separation in regulated cell death of plants

PCD in plants is a spontaneous process that occurs during growth, development, and environmental adaptation. It plays crucial roles in plant development by inducing cell death to remodel plant structures and activating a cascade of stress-responsive genes, which is vital for adaptation and survival under adverse conditions [111]. However, the role of LLPS in this context appears to be focused primarily on immunity-associated cell death, particularly the hypersensitive response (HR) and the tightly coupled process of death threshold control linked to ROS homeostasis, as detailed below.

Zavaliev et al. revealed that salicylic acid signaling induces the formation of nonexpressor of pathogenesis-related genes 1 (NPR1) condensates, which recruit components including NLR immune receptors, stress-responsive molecules, and proteostasis-related proteins, thereby promoting the ubiquitination and degradation of specific substrates, enhancing cell survival and fine-tuning the magnitude of effector-triggered immunity (ETI)-associated cell death [112]. In separate studies, Song, Lapin, and colleagues demonstrated that condensation of TIR domain-containing proteins elucidates LLPS-mediated promotion of plant RCD: upon pathogen recognition, upstream TIR proteins form condensates that scaffold and amplify enzymatic activity, with the signal being subsequently relayed to the EDS1-associated death module, thereby driving the execution of hypersensitive response (HR)-type RCD [113, 114]. These findings indicate that biomolecular condensates can function not only as pro-death signaling platforms but also as death-restricting mechanisms, thereby critically shaping plant cell fate. Beyond direct evidence at the level of immune receptors, the link between LLPS and plant RCD is further exemplified by its role in ROS-mediated RCD regulation: in Arabidopsis, catalase 2 (CAT2) is recruited in a redox-dependent manner into phase-separated condensates formed by Lesion Simulating Disease 1 (LSD1) [115]. This dynamic condensation enables precise control of CAT2 localization between peroxisomes and the nucleus, along with modulation of its enzymatic activity, thereby fine-tuning the spatiotemporal distribution of H₂O₂ and regulating key downstream execution nodes of cell death. Such a mechanism ensures that RCD signaling is spatially confined to infected or damaged tissues, preventing excessive propagation of cell death [115, 116]. In summary, the current understanding of the “LLPS–plant RCD” relationship can be framed as follows: LLPS serves as an upstream spatiotemporal organizational principle in regulating plant cell death, whose core function lies in integrating immune recognition and ROS signaling to determine whether the cell crosses the death threshold. Notably, plants and animals exhibit significant differences in the hierarchical action of LLPS within RCD pathways. In animal systems, LLPS-driven higher-order assemblies operate not only at the level of upstream signal sensing but also penetrate into the execution layer—as seen in the NLRP3–ASC speck during pyroptosis or the assembly of the DISC in apoptosis. By contrast, the involvement of LLPS in further downstream cascading signaling within plant RCD remains largely unexplored. Therefore, establishing direct causal links between LLPS and more terminal execution steps—such as ferroptosis-like death and developmental PCD—represents a critical frontier for future research.

## Potential liquid–liquid phase separation-related factors in regulated cell death

In addition to the established role of LLPS in RCD, we also surveyed molecules and molecular families with the potential to undergo LLPS and regulate RCD. These include SGs, the long non-coding RNAs (lncRNAs) family, Tau, and the DDX protein family.

### SGs

SGs contain RNA-binding proteins, translationally stalled mRNAs, translation initiation factors, and various other proteins that assemble through LLPS of the aforementioned proteins and play a role in regulating protein translation [117]. In addition to the aforementioned interactions, SGs exert regulatory functions in other forms of RCD. Specifically, in ferroptosis, G3BP1, a cytoplasmic stress granule protein that responds to oxidative stress, can attenuate cellular sensitivity to ferroptosis, thereby reducing its incidence [118]. Furthermore, in autophagy, the studies have confirmed that SGs play a regulatory role. For instance, TDP43(an SG assembly-related protein [119]) has been shown to modulate the autophagic process [120]. Additionally, ELAVL1(another SG-associated molecular [121]) has been demonstrated to correlate with the expression levels of autophagy-related proteins [122]. The above studies have confirmed the involvement of SGs in various cell death processes. Although the direct role of LLPS in these phenomena has not yet been fully elucidated, given that SGs are classic phase-separated condensates, it is anticipated that further research will uncover novel molecular mechanisms by which LLPS SG influences cell death.

### Long non-coding RNA

LncRNAs, owing to their IDRs, can function as scaffolds to drive LLPS and facilitate the formation of protein condensates, which exert regulatory functions in RCD [123].

During apoptosis, LINC00618, a member of the lncRNA family, promotes apoptosis by upregulating the expression levels of BAX and caspase-3 [124]; Termed TP53-inhibiting lncRNA (TILR), this long non-coding RNA maintains p53 transcriptional activity at a sufficiently low level to prevent the occurrence of apoptosis [125]； The long non-coding RNA lnc-CCNL1-3:1 (CCNL) induces apoptosis by increasing the concentration of reactive oxygen species (ROS) via its interaction with FOXO1 [126]; Upon activation by FOXO3a, LINC01480 inhibits the PI3K/AKT signaling pathway, thereby inducing apoptosis [127]; LINC5438 inhibits apoptosis through the suppression of the mitochondrial apoptotic pathway [128].

In the context of autophagy, lncRNA-H19 promotes development of autophagy by upregulating the PI3K/AKT/mTOR signaling pathways [129]; lncRNA GAS5 regulates the expression of ATG5 and ATG12 through its interaction with miR-181c-5p and miR-1192, thereby promoting autophagy [130]; Long non-coding RNA MEG3 has been demonstrated to promote the expression of the autophagy-related gene ATG7 by binding and inhibiting miR-181c-5p, thereby mediating autophagy. Conversely, it can also inhibit autophagy by downregulating the AKT/TSC/mTOR signaling pathway [130, 131]; Long non-coding RNA LINC01207 promotes autophagy by sequestering miR-143-5p, thereby inhibiting its expression and upregulating AGR2 expression [132]; lncRNA H19 inhibits autophagy via the DUSP5-ERK1/2 signaling axis [133]; lncRNA-GBCDR upregulates ATG5-ATG12 expression and activates autophagy by interacting with PGK1 and preventing its degradation [134]; Long non-coding RNA NEAT1 inhibits autophagy by activating AMPK, which indirectly mediates the phosphorylation of downstream mTOR protein [135].

Under hypoxic conditions, LncRNA HABON inhibits the phosphorylation of RIPK1 and MLKL, and interacts with VDAC1 on the mitochondria, thereby suppressing the opening of the mitochondrial permeability transition pore (mPTP) and inhibiting necroptosis [136]; LncRNA-107053293 functions as a ceRNA (competitive endogenous RNA) for miR-148a-3p in chicken respiratory epithelium, thereby regulating ammonia-induced necroptosis mediated by the miR-148a-3p target FAF1 [137]; LncRNA CRLA inhibits RIPK1-induced necroptosis by binding to the middle domain of RIPK1, thereby impairing the interaction between RIPK1 and RIPK3 [138].

In pyroptosis, lnc RNA H19 acts as a sponge for miR-138-5p by competitively binding to rno-miR-138-5p, thereby upregulating NLRP3 expression and promoting NLRP3-mediated pyroptosis. Additionally, studies have reported that H19 can inhibit ox-LDL-induced activation of the nuclear factor-kappa B (NF-κB) pathway, suppress cytokine release, and prevent caspase-1-dependent pyroptosis [139–141]; similar mechanisms are observed in LncRNA MIR155HG and LncRNA GAS5, which function as ceRNAs to sequester miR-223-3p, thereby promoting NLRP3 expression and mediating pyroptosis [142, 143]; LncRNA MIR22HG mediates the regulation of miR-9-3p on IGF1, thereby promoting autophagy and inhibiting pyroptosis in cells [144].

In ferroptosis, LINC00618 promotes ferroptosis by downregulating SLC7A11 expression and increasing intracellular iron ion concentration [124]. Additionally, above NEAT1 enhances the direct binding of Slc7a11, thereby promoting GPX-4 activity and inhibiting ferroptosis [135].

In conclusion, lncRNAs play a broad and critical regulatory role in RCD (Table 1). The widely accepted theory posits that lncRNAs function as molecular scaffolds, providing binding sites for other molecules, which facilitates LLPS and the formation of membraneless organelles [145]. Consequently, investigating the LLPS phenomena regulated by additional lncRNAs during RCD may not only broaden our understanding of the extensive roles of LLPS but also enhance our insights into the mechanisms underlying RCD.

**Table 1:** The functions of the remaining Long non-coding RNA in RCD.

	Apoptosis	Autophagy	Necroptosis	Pyroptosis	Ferroptosis	References
Lnc RNA	LINC00618 upregulates the expression levels of BAX and caspase-3 TILR inhibits p53 transcriptional activity CCNL interacts with FOXO1, increasing the concentration of ROS LINC01480 inhibits the PI3K/AKT signaling pathway LINC5438 inhibits the suppression of the mitochondrial apoptotic pathway	H19 regulates the PI3K/AKT/mTOR signaling pathways and DUSP5-ERK1/2 signaling axis GAS5 regulates the expression of ATG5 and ATG12 MEG3 promotes the expression of the autophagy-related gene ATG7 LINC0120 inhibits miR-143-5p and upregulates AGR2 GBCDR upregulates ATG5 ATG12 expression and preventing PGK1 degradation NEAT1 activates AMPK to indirectly mediate the phosphorylation of the downstream mTOR protein	HABON inhibits the phosphorylation of RIPK1 and MLKL and interacts with VDAC1 on the mitochondria LncRNA-107053293 as a competitive endogenous RNA for miR-148a-3p to regulate ammonia-induced necroptosis CRLA binds to the middle domain of RIPK1 to impair the interaction between RIPK1 and RIPK3	H19 upregulates NLRP3 expression and inhibits ox-LDL-induced activation of the NF-κB pathway MIR155HG and GAS5 sequesters miR-223-3p to promote NLRP3 expression MIR22HG mediates the regulation of miR-9-3p on IGF1 to inhibit pyroptosis	LINC00618 downregulates SLC7A11 expression and increases intracellular iron ion concentration NEAT1 enhances the direct binding of Slc7a11, to promote GPX4 activity	[124] [125] [126] [127] [128] [129] [130] [131] [132] [133] [134] [135] [136] [137] [138] [139] [140] [141] [142] [143] [144] [145]

### Tau

Tau, a microtubule-associated protein extensively distributed throughout the central nervous system (CNS), exhibits a propensity for aggregation into clusters [146]. In addition to its established role in apoptosis, recent evidence has confirmed Tau’s involvement in regulating other RCD.

In the context of autophagy, Tau protein aggregation has been shown to activate CCAAT enhancer binding protein beta (CEBPB), which stimulates the interaction between acidic nuclear phosphoprotein 32 family member A (ANP32A) and SET, leading to the formation of the inhibitor of histone acetyltransferase (IHNAT). Consequently, IHNAT inhibits histone acetylation by histone acetyltransferase (HAT) and suppresses the transcription of IST1, a factor associated with ESCRT-II and involved in ESCRT-III function. This ultimately results in reduced formation of the endosomal sorting complexes required for transport (ESCRT), which are essential for autophagosome-lysosome fusion [147]. Furthermore, several studies have demonstrated that both phosphorylated and acetylated Tau proteins can inhibit the progression of autophagy [148, 149].

In the context of necroptosis, pathological Tau protein promotes the formation of granulovacuolar neurodegeneration vesicles (GVDs) enriched in RIPK1, RIPK3, and phosphorylated MLKL. These GVDs function as critical checkpoints in the process of programmed necrosis [150]. Furthermore, pathological tau can directly upregulate the phosphorylation of RIPK1, RIPK3, and MLKL, and promote the assembly of the RIPK1/RIPK3/MLKL necrosome, thereby directly activating the necroptotic pathway [151].

In the context of pyroptosis, studies have confirmed that pathological Tau can upregulate the NLRP3/caspase-1/GSDMD pathway, thereby promoting the induction of pyroptosis [152].

In the context of ferroptosis, studies have demonstrated that lactoylation at the K677 site of tau protein promotes p38 MAPK-mediated ferritinophagy, thereby inducing iron ion release and ultimately leading to ferroptosis [153]. Furthermore, several studies have confirmed that the overexpression and hyperphosphorylation of Tau can lead to iron overload, thereby inducing ferroptosis [154]. Additionally, the 14-3-3/CAMK2D/Tau complex can promote abnormal phosphorylation and aggregation of the Tau protein, leading to the accumulation of lipid peroxides, thereby facilitating the onset of ferroptosis [155].

The above content outlines additional specific roles of the Tau protein in RCD (Table 2). As previously discussed, the Tau protein exhibits the aggregation capacity for LLPS. The aggregation of Tau protein and its phosphorylated forms contributes not only to the formation of neurofibrillary tangles but also to selective neuronal loss and reduced glucose metabolic efficiency [156, 157]. Consequently, further investigation into how the Tau protein’s LLPS behavior influences RCD is likely to enhance our understanding of the pathological mechanisms underlying Alzheimer’s disease.

**Table 2:** The remaining functions of Tau in RCD.

	Autophagy	Necroptosis	Pyroptosis	Ferroptosis	References
**Tau**	Tau protein promotes the formation of IHNAT to inhibit histone acetylation and suppress the transcription of IST1, thereby reducing the formation of ESCRT Phosphorylated and acetylated Tau proteins can inhibit the progression of autophagy	Pathological Tau protein promotes the formation of GVDs, functioning as critical checkpoints in the process of programmed necrosis pathological tau directly upregulates the phosphorylation of RIPK1, RIPK3, and MLKL, and promote the assembly of the RIPK1/RIPK3/MLKL necrosome	Pathological Tau upregulates the NLRP3/caspase-1/GSDMD pathway	Lactoylation at the K677 site of tau protein promotes p38 MAPK-mediated ferritinophagy to induce iron ion release Overexpression and hyperphosphorylation of Tau lead to iron overload the 14-3-3/CAMK2D/Tau complex promotes the accumulation of lipid peroxides	[147] [148] [149] [150] [151] [152] [153] [154] [155]

### DDX family protein

The DDX family protein, characterized by its IDRs, has been demonstrated to possess the capability for LLPS and plays a crucial role in a variety of biological processes, including RNA transcription and translation, immune signal transduction, and energy substance metabolism [158, 159]. In addition to its role in the aforementioned pyroptosis, the DDX protein also plays a significant role in other forms of RCD.

During apoptosis, DDX3X interacts with GSK3 and CIAP1 to form a complex that competes with death ligands for binding to death receptors, thereby inhibiting the activation of these receptors and suppressing extrinsic apoptotic signaling [160]. DDX47 has been demonstrated to interact with GABA(A) receptor-associated protein (GABARAP), thereby modulating apoptosis [161]. RIG-I (DDX58) interacts with MDA-5 and utilizes the mitochondrial adapter protein Cardif (IPS-1) to upregulate the expression of BH3-only proteins Puma and Noxa, thereby promoting intrinsic apoptosis in melanoma cells [162].

In ferroptosis, DHX56 binds to GPX4 mRNA and subsequently recruits the m^6^A reader YT521-B homology domain containing 2 (YTHDC2), thereby facilitating methylation-dependent enhancement of GPX4 expression, ultimately inhibiting ferroptosis [163].

In pyroptosis, RIG-I (DDX58) functions as an RNA sensor to recognize viral RNA, subsequently interacting with ASC and CARD proteins to assemble into an inflammasome [164]. DHX9 and DHX33 can also recognize double-stranded RNA, thereby promoting the assembly of inflammasomes [165, 166].

In summary, we have systematically reviewed the diverse roles of DDX family members in RCD (Table 3). LLPS, a hallmark behavior of the DDX family [158], plays roles in various biological processes, including tumor regulation, immune signal transduction, and cell proliferation [18, 167, 168]. Consequently, further investigation into the LLPS behavior of DDX family members during RCD is likely to deepen our understanding of cellular physiology and pathology and facilitate the development of novel therapeutic strategies.

**Table 3:** The functions of the remaining DDX family members in RCD.

	Apoptosis	Pyroptosis	Ferroptosis	References
DDX Family	DDX3X/GSK3/CIAP1 complex competes with death ligands for binding to death receptors DDX47 interacts with GABA(A) receptor-associated protein (GABARAP), thereby modulating apoptosis DDX58 interacts with MDA-5 and utilizes the mitochondrial adapter protein Cardif to upregulate the expression of BH3-only proteins Puma and Noxa	DDX58 functions as an RNA sensor to recognize viral RNA, subsequently interacting with ASC and CARD proteins to assemble into an inflammasome DHX9 and DHX33 recognize double-stranded RNA, thereby promoting the assembly of inflammasomes	DHX56 binds to mGPX4 and subsequently recruits the m6A reader YT521-B homology domain containing 2 to facilitate GPX4 expression	[160] [161] [162] [163] [164] [165] [166]

## The LLPS of RCD in diseases

In the preceding discussion, we have elucidated the regulatory mechanisms by which LLPS governs RCD, highlighting its pivotal role in this process: LLPS modulates the functionality of signaling molecules across multiple RCD pathways, including BCL-2 protein, iron ions, and GPX4 mRNA. By silencing or activating these pivotal determinants, LLPS ultimately governs cellular fate [25, 96, 109]. Under physiological conditions, RCD serves as a critical guardian of cellular homeostasis and exerts protective functions. However, in pathological contexts, dysregulated LLPS can drive aberrant RCD, contributing significantly to disease initiation and progression. To deepen our understanding of the interplay between RCD and disease pathogenesis, we present a phase separation-centered perspective that not only clarifies this mechanistic link but also offers novel insights for therapeutic intervention.

### Tumors

Tumor, one of the leading causes of human mortality, has seen an increasing number of underlying developmental mechanisms uncovered in recent years. LLPS and RCD, as emerging focal points in cancer research, have been widely shown to engage in a synergistic interplay during tumorigenesis and progression. Therapeutic strategies specifically designed to target this LLPS-RCD axis hold great promise for providing novel and effective approaches in the fight against cancer. For example, in breast cancer, the ligand XS561 promotes the LLPS of Nur77, leading to condensate formation with Bcl-2, which subsequently induces apoptosis in triple-negative breast cancer cells and exerts inhibitory effects on MCF-7/LCC2 tamoxifen-resistant breast cancer (TAMR) [25]. In triple-negative breast cancer (TNBC), lncFASA undergoes LLPS with PRDX1, disrupting PRDX1-mediated regulation of the SLC7A11-GPX4 axis, leading to the accumulation of lipid peroxides and triggers ferroptosis, thereby suppressing TNBC progression [95]. These findings provide novel mechanistic insights into potential therapeutic strategies for breast cancer. The same family member RUNX1-IT1 interacts with IGF2BP1 to undergo LLPS, resulting in enhanced formation of IGF2BP1 condensates that bind to and stabilize GPX4 mRNA, which suppressing ferroptosis and promotes breast cancer progression [96]. This study reveals the RUNX1-IT1/IGF2BP1/GPX4 regulatory axis, highlighting its potential as a therapeutic target and offering a promising strategy for the treatment of breast cancer patients. In the regulation of transcription and translation, TGF-β promotes the synthesis of Samd2/3/4 and facilitates their nuclear translocation, leading to stabilization of TAT expression and subsequent induction of apoptosis in hepatocellular carcinoma cells [40]. These findings not only enhance our understanding of the molecular mechanisms underlying hepatocellular carcinoma progression but also provide novel insights into potential therapeutic strategies for this malignancy. Another key transcriptional regulatory factor, YAP/TAZ, undergoes phase separation under the stimulation of Mn^2+^, thereby entering the nucleus to promote the expression of ACSL4, leading to the peroxidation of polyunsaturated fatty acids, thereby exacerbating ferroptosis and inhibiting the occurrence of oral squamous cell carcinoma from the perspective of ion regulation [97]. The above findings highlight the critical role of phase separation in the transcriptional regulation of RCD. While epigenetics has emerged as a prominent research area, the involvement of phase separation in epigenetic regulation remains incompletely understood. Nevertheless, leveraging the biophysical properties of phase separation to modulate RCD-related diseases through epigenetic mechanisms represents a promising and potentially transformative therapeutic strategy. In addition, p53, a well-known tumor suppressor protein, interacts with 53BP1, which undergoes self-phase separation in the absence of AHNAK. The resulting biomolecular condensates recruit p53 and enhance its transcriptional activity, thereby promoting apoptosis in tumor cells [43]. This mechanism provides a theoretical foundation for the development of p53-targeted anti-cancer therapies. On the other hand, during tumor chemotherapy, oxidative stress induces Z-RNA formation, triggering stress granule phase separation and the assembly of biomolecular condensates. These condensates recruit Z-RNA, facilitating its recognition by ZBP1 within the compartments, which in turn activates downstream necroptotic signaling pathways and enhances the therapeutic efficacy of chemotherapy [56]. In the context of crosstalk among cell death pathways, PRMT6 promotes p62 oligomerization, thereby driving its LLPS and the formation of p62 condensates, which recruit and sequester Keap1, facilitating its degradation and consequently inhibiting Keap1-mediated Nrf2 turnover, leading to suppression of ferroptosis [105]. The finding highlights that targeting PRMT6-mediated ADMA on p62 may represent a novel therapeutic strategy to modulate ferroptosis sensitivity in cancer treatment. The above-mentioned research confirmed the promoting mechanism and inhibitory effect of RCD regulated by phase separation in tumors, demonstrating that LLPS can serve as a regulator of RCD, thereby inhibiting tumor progression. In response to this phase separation, research has developed icFSP1, which, by phase separating from FSP1 (a parallel pathway to GXP4), forms aggregates that inhibit the function of FSP1, reduce its affinity for membranes, decrease ubiquinone and vitamin K, and ultimately induce ferroptosis in tumor cells [101]. This result not only confirms the feasibility of treating diseases by inducing phase separation of key RCD factors but also provides the idea of using phase separation as a molecular switch, indicating that the diversity and explorability of phase separation in RCD for disease treatment still require extensive research to fill the gap in this area.

### Neurological diseases

Neurological diseases, among the most complex disorders in the human body, are characterized by underlying pathological mechanisms involving protein misfolding and aggregation. Among neurodegenerative diseases, Alzheimer’s disease is characterized by a high clinical incidence, and recent studies have demonstrated that Tau-441, a key isoform of the Tau protein, undergoes LLPS to form condensates that recruit hexokinase, thereby inhibiting the competitive binding of hexokinase and Bax to VDAC1; more importantly, the study demonstrates that magnetic fields can disrupt phase-separated condensates, thereby inhibiting apoptosis [39]. This finding provides novel mechanistic insights into Alzheimer’s disease pathogenesis from the perspective of RCD with LLPS, and also suggests that modulating phase-separated condensates through external interventions may serve as an effective therapeutic strategy for diseases associated with RCD. In addition, neuronal protein aggregation exacerbates neuroinflammation and contributes to inflammation-mediated neurotoxicity through the induction of pyroptosis. Specifically, aggregated p62 recruits DDX6 and promotes LLPS, leading to the formation of pd-pBs. These condensates act as scaffolding platforms that recruit APC components within the inflammasome complex, facilitating the assembly of mature inflammasomes [85]. The activation of this pathway enhances neuroinflammatory responses via pyroptosis, thereby promoting the progression of neurodegenerative diseases.

### Cardiovascular diseases and respiratory system diseases

Cardiovascular diseases, as a major class of high-risk disorders, significantly impair patient survival and quality of life. Among these, myocardial infarction is one of the leading causes of global morbidity and mortality, and the subsequent ischemia-reperfusion injury exacerbates myocardial damage—a process involving pyroptosis. Emerging evidence suggests that phase separation plays a role in this pathological cascade by leveraging PGAM5 as a molecular scaffold to drive the condensation of MAVS, thereby promoting NLRP3 inflammasome activation and ultimately triggering pyroptotic cell death, which contributes to I/R injury [84]. With respect to respiratory diseases, the novel coronavirus (SARS-CoV-2) has caused a global pandemic and poses a significant threat to public health worldwide. Viral replication and transcription are closely linked to phase separation and pyroptosis. Specifically, the nucleocapsid (N) protein undergoes LLPS with TAK1 and IKK, while simultaneously suppressing stress granule formation. This dual action promotes viral replication and assembly, activates the NF-κB signaling pathway, and triggers pyroptosis, thereby exacerbating the systemic cytokine storm and ultimately impairing immune function in patients with COVID-19 [89]. The common mechanisms of the above two diseases reveal that phase separation couples pathogen recognition, inflammatory signaling, and pyroptosis effects through scaffold-like assembly, constituting a cross-disease immunopathological regulatory paradigm. Targeting the condensed state of the phase separation system or the interaction interfaces of key scaffold proteins is expected to provide new intervention strategies for cardiovascular emergencies and severe respiratory infections.

## Conclusion and prospects

LLPS, as an emerging mechanism of molecular interaction, has become a focal point in biology. In this review, we summarize the fundamental conditions for LLPS formation and the key characteristics of condensates, while highlighting their function in various biological processes [169]. We further dissect the involvement of LLPS in RCD mechanisms, specifically in the contributions of LLPS to apoptosis, necroptosis, autophagy-dependent cell death, pyroptosis, and ferroptosis. This effect not only functions as a molecular interaction mechanism to facilitate cell death but also exerts an isolating effect on key molecules in the death pathway, thereby suppressing the progression of cell death. However, research on LLPS-related RCD remains incomplete due to significant gaps. One major limitation lies in the challenges of defining LLPS condensates with precise criteria and characterizing their internal organization and structure using advanced techniques [12]. Currently, predicting the LLPS potential of molecules primarily depends on IDRs, yet studies have shown that IDRs alone are insufficient to guarantee LLPS [17]. Additionally, experimental methods for detecting LLPS, such as droplet morphology observation, fluorescence recovery after photobleaching (FRAP), and the use of LLPS inhibitors (e.g., 1,6-hexanediol), lack a universally accepted “gold standard”. Another critical factor is that investigations into the interplay between LLPS and RCD are still in their early stages, with limited literature available to support in-depth exploration. To address these gaps, we propose the following four key recommendations: (1) Construct phase-separation-defective but function-preserved separation mutants to directly target the essence of phase separation, demonstrating that altering phase separation behavior itself can determine cell death fate; (2) Analyze the continuum of “droplet-gel-fibril” state transitions—given the highly dynamic nature of condensates in RCD, particularly the compaction of NLRP3 into inflammasomes and the ordered oligomerization and filamentous assembly in apoptosis—to infer material-state changes and predict phase separation involvement; (3) Develop genuine phase transition modulators that selectively alter condensation thresholds, component partitioning, or droplet-to-ordered assembly transitions, rather than indiscriminately dissolving condensates; (4) Methodologically, avoid relying solely on 1,6-hexanediol as definitive evidence for LLPS, as it directly inhibits multiple kinases and phosphatases, which may confound RCD studies given their dependence on enzymatic activity and phosphorylation cascades. Integrating these four aspects, investigating LLPS in RCD should incorporate genetic, biophysical, functional rescue, and time-resolved imaging approaches as core criteria.

As a critical component of cellular behavior, RCD encompasses a wide range of death modalities, including cuproptosis, disulfidptosis, entotic cell death, netotic cell death, parthanatos, lysosome-dependent cell death, alkaliptosis, and oxeiptosis. Abnormal RCD can serve as a key driver for various diseases, such as cancer, neurodegenerative disorders, and autoimmune diseases. Moreover, abnormal LLPS is recognized as one of the contributing factors to disruptions in physiological homeostasis. Therefore, investigating LLPS in the context of abnormal RCD not only sheds light on the mechanisms underlying disease onset and progression caused by RCD but also offers potential avenues for refining currently available drugs targeting RCD. Our review indicates that the occurrence of LLPS in RCD is influenced by multiple factors, such as local molecular concentration, intermolecular adhesion and affinity, activation of chaperone molecules, and environmental conditions. Additionally, studies on LLPS have demonstrated that its occurrence can be modulated in vitro by manipulating the conditions that promote or inhibit LLPS [20]. This suggests potential strategies for targeting the conditions that govern LLPS, such as modulating local protein concentrations, computationally simulating chaperone proteins, or altering the osmotic pressure and pH of the local environment, to design drugs aimed at regulating RCD. Meanwhile, identifying specific LLPS-related vulnerable nodes in RCD could facilitate the discovery of therapeutic targets for RCD-associated diseases. For instance, in pyroptosis, LLPS has emerged as an organizational principle for inflammasome assembly and related immune signaling [170]. From a drug development perspective, targeting the transition from droplet-state sensors to ordered inflammasome assemblies—by raising the energy barrier for this conversion—may potentially suppress pathological pyroptosis while preserving host defense functions. Additionally, phase separation of SGs mentioned earlier serves as a molecular switch determining cell fate across multiple RCD pathways [50]. Identifying molecules that modulate the liquid dynamics of SGs and leveraging their hub function in cross-regulating cell death signaling may enable more efficient suppression of disease progression.

In conclusion, constructing separation mutants that specifically alter phase behavior without disrupting protein function, developing genuine “condensate modulators” capable of modifying transition thresholds and state conversions, identifying LLPS vulnerabilities within RCD, and uncovering key hub molecules across multiple cell death pathways—these strategies will not only help elucidate the biophysical principles of phase separation in RCD but also provide highly specific potential therapeutic avenues for intervening in RCD-related diseases.
